# Supplementary material for: Human cytomegalovirus-encoded G protein-coupled receptor (GPCR) UL78 regulates viral reactivation
Source: J Virol. 2025 Aug 27;99(9):e01057-25. doi: 10.1128/jvi.01057-25 (PMC12455920; doi:10.1128/jvi.01057-25)
Supplement: Supplemental material — Figures S1 to S5; Tables S1 and S2. [file jvi.01057-25-s0001.pdf]

Supplemental Figures

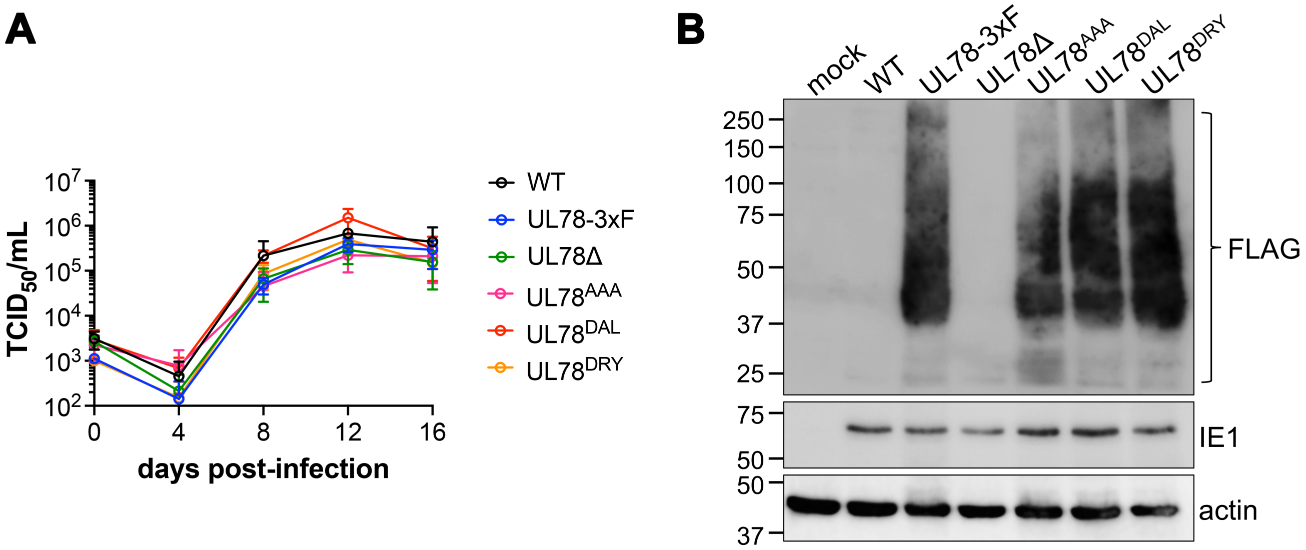

Figure S1. Recombinant and deletion UL78 viruses display wild type lytic replication kinetics in fibroblasts. (A) Fibroblasts (MRC-5) were infected with the indicated viruses, supernatants collected over a 16-day time course and then analyzed by $TCID_{50}$ assay. N=3 biological replicates, each with 3 technical replicates for each virus at each time point. Error bars represent SD of the mean; significance calculated using t-test. Data is not significant. **(B)** Fibroblasts (MRC-5) were mock-, WT-, UL78-3xF, UL78Δ-, $UL78^{AAA}$-, $UL78^{DAL}$-, or $UL78^{DRY}$-infected (MOI = 0.5 $TCID_{50}$/cell). Lysates were collected 96 hpi and probed for FLAG (to detect UL78), IE1, or actin (loading control). N=3, representative blots shown.

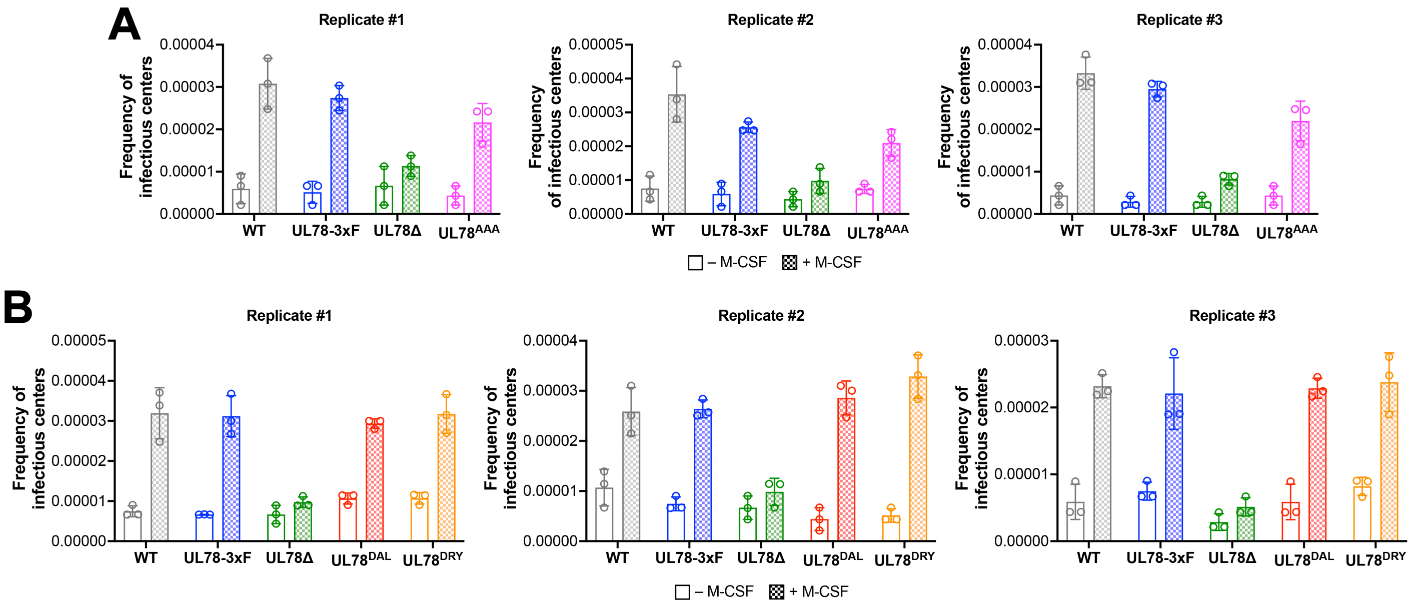

Figure S2. Each independent replicate for combined ELDA data in Figure 2. (A) Data corresponds to Fig. 2A. **(B)** Data corresponds to Fig. 2B. **(A,B)** Primary $CD14^+$ cells were infected with the indicated viruses at MOI = 1.0 $TCID_{50}$/cell and cultured for 7 d in conditions favoring latency. $CD14^+$ cells were maintained under latent conditions (- M-CSF) or treated with M-CSF (+ M-CSF) to differentiate the cells. Cells were then co-cultured with naïve fibroblasts, and following 14 d in co-culture, ELDA was used to quantify reactivation. Data points (open circles) represent technical replicates. Error bars represent SD of the mean.

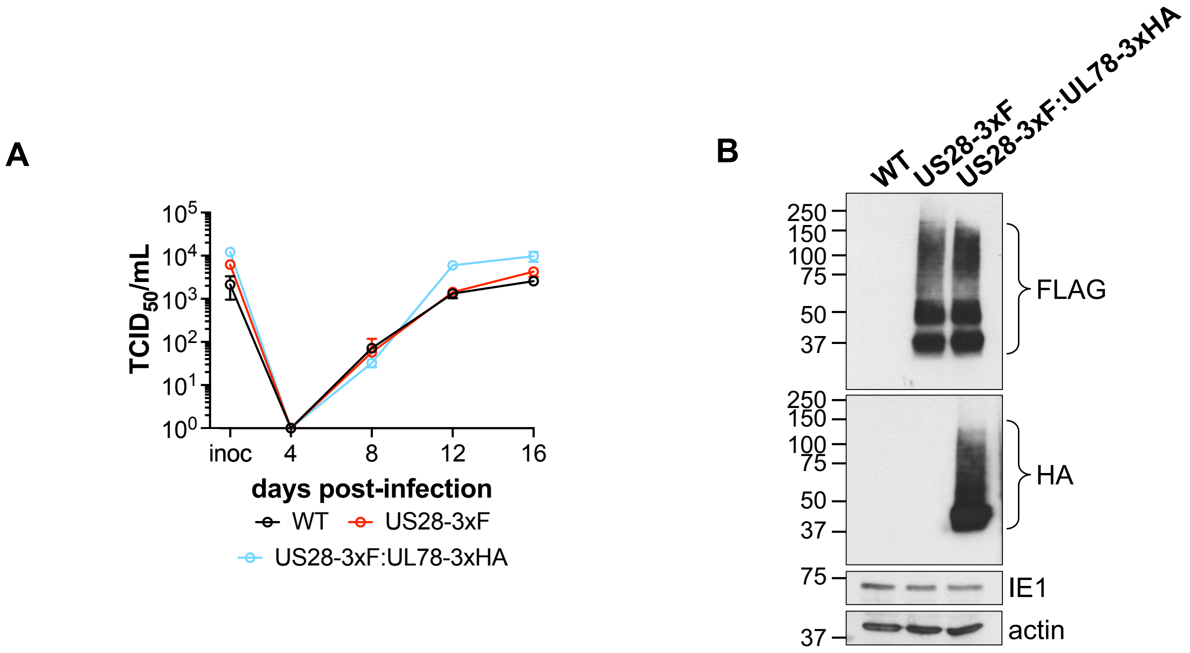

Figure S3. TB40/E*mCherry*-US28-3xF:UL78-3xHA (US28-3xF:UL78-3xHA) infection of fibroblasts displays wild type growth kinetics. (A) Fibroblasts (NuFF-1) were infected with the indicated viruses (MOI = 0.01 $TCID_{50}$/cell). Supernatants were collected at the indicated time points and titers assessed by $TCID_{50}$ assay. N=2 biological replicates; each with three technical replicates per condition. Representative assay shown. Error bars represent SD of the mean. **(B)** Fibroblasts (NuFF-1) were infected with the indicated viruses (MOI = 0.5 $TCID_{50}$/cell) for 96 h. Lysates were collected and probed for FLAG (to detect US28), HA (to detect UL78), IE1, and actin. N=2; representative blots shown.

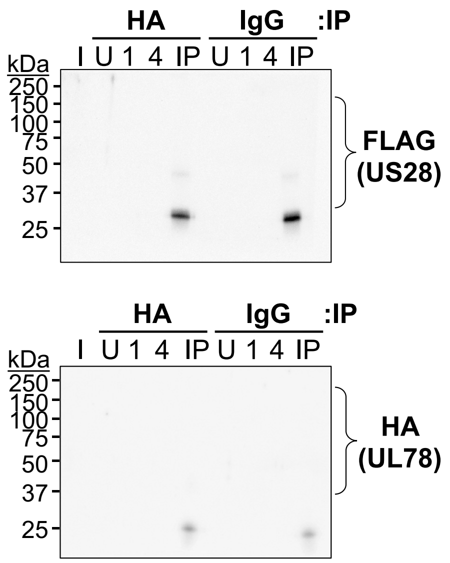

Figure S4. TB40/E*mCherry* control data for Figure 4. NuFF-1 fibroblasts were infected (MOI = 0.5 $TCID_{50}$/cell) with TB40/E*mCherry.* Cells were harvested 96 hpi and lysates were immunoprecipitated (IP) with either anti-HA or anti-IgG antibodies and immunoblotted for (**top**) FLAG to detect US28 or (**bottom**) HA to detect UL78**.** I, input; U, unbound; 1, 1st wash; 4, 4th wash; IP, immunoprecipitated sample. Representative blots shown.

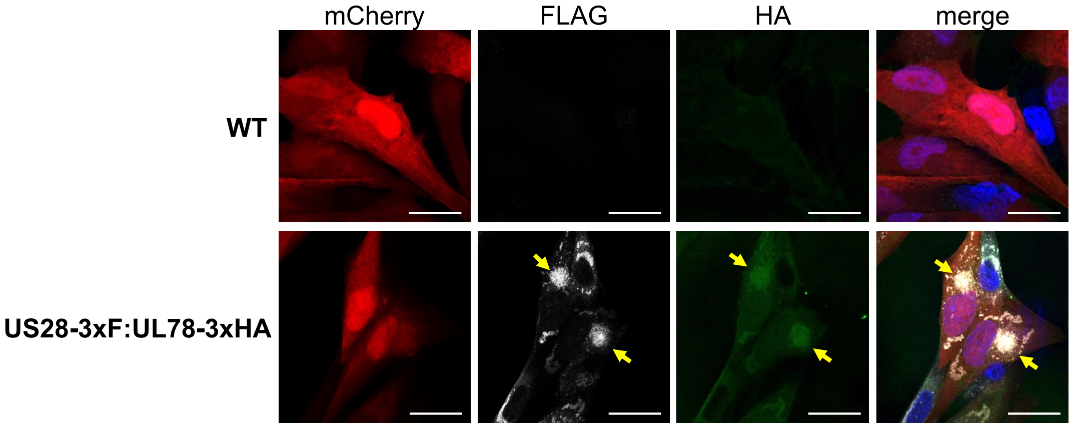

Figure S5. UL78 and US28 colocalize in the VAC in lytically infected fibroblasts. NuFF-1 fibroblasts were infected with either TB40/E*mCherry* (wild type, WT) or TB40/E*mCherry*-US28-3xF:UL78-3xHA (MOI = 0.5 $TCID_{50}$/cell) for 96 h. Cells were fixed, permeabilized, and then stained for US28 with antibodies directed at the triple FLAG epitope (white) and UL78 with antibodies directed at the triple HA epitope (green). mCherry (red) serves as a marker of infection and DAPI (blue) was used to visualize cell nuclei. Yellow arrows denote areas of US28 and UL78 co-localization. Images were acquired using a 63x objective; scale bars, 50µm. N= 2; representative images shown.

Table S1. Oligonucleotides used to generate viral recombinants.

Primer use	Sequence*	Target**
Generating galK insertion (for UL78^{AAA}, UL78^{DAL}, UL78^{DRY})	CGACGTGGGCCTATATTCGACGGCGTTGTTTTTCCTCTTTCTGATACTGGCCTGTTGACAATTAATCATCGGCA	UL78 galK For
	GTTTTCGCGCGTCTCATGATGCCAGAGATCACGGCCGTAAGATATGGCCGTCAGCACTGTCCTGCTCCTT	UL78 galK Rev
UL78^{AAA} insert	TCTTTGTCGACGACGTGGGCCTATATTCGACGGCGTTGTTTTTCCTCTTTCTGATACTGGCAGCTGCGTCGGCCATATCTTACGGCCGTGATCTCTGGCATCATGAGACGCGCGAAAACGCCGGCGTGGCG	UL78^{AAA} gBlock
UL78^{DAL} insert	TCTTTGTCGACGACGTGGGCCTATATTCGACGGCGTTGTTTTTCCTCTTTCTGATACTGGATGCACTGTCGGCCATATCTTACGGCCGTGATCTCTGGCATCATGAGACGCGCGAAAACGCCGGCGTGGCG	UL78^{DAL} gBlock
UL78^{DRY} insert	TCTTTGTCGACGACGTGGGCCTATATTCGACGGCGTTGTTTTTCCTCTTTCTGATACTGGATCGTTACTCGGCCATATCTTACGGCCGTGATCTCTGGCATCATGAGACGCGCGAAAACGCCGGCGTGGCG	UL78^{DRY} gBlock
UL78 gBlock amplification primers	TCTTTGTCGACGACGTGGGC	UL78 gBlock For
	CGCCACGCCGGCGTTTTCGC	UL78 gBlock Rev
Generating galK insertion (for UL78-3xHA)	GCACCGACGGCGAAAACACCGTCGCGTCCGACGCAACGGTGACGGCATTACCTGTTGACAATTAATCATCGGCA	UL78-3xHA galK For
	GTGATTTATCTGCCACTTTTCTCCCCGCTGCCGTACAGCGCCGCCGCTCATCAGCACTGTCCTGCTCCTT	UL78-3xHA galK Rev
UL78-3xHA insert	GCACCGACGGCGAAAACACCGTCGCGTCCGACGCAACGGTGACGGCATTATACCCATATGACGTTCCAGACTACGCGTATCCGTACGACGTTCCGGATTACGCTTACCCTTACGACGTACCTGACTACGCTTGAGCGGCGGCGCTGTACGGCAGCGGGGAGAAAAGTGGCAGATAAATCAC	UL78-3xHA gBlock
UL78-3xHA gBlock amplification primers	TGCACCGACGGCGAAAACACCG	UL78-3xHA gBlock For
	GTGATTTATCTGCCACTTTTCTCC	UL78-3xHA gBlock Rev
Sequencing primers UL78	GCTGCATAAAGGGCGAACG	UL78 seq For
	TGGCTAACGACGTGTGAACC	UL78 seq Rev

*Primer sequences shown the 5’ to 3’ orientation. Underlined sequences correspond to the pGalK plasmid. **For, forward; Rev, reverse.

Table S2. Oligonucleotide pairs used for PCR.

Target	Forward (5’- 3’)*	Reverse (5’-3’)*
UL69	GGCTGATGATCTTGCGGGAA	CGAGAGTCTACGTCTGGCAC
MDM2	CCCCTTCCATCACATTGCA	ACCCACTCCTCCACCTTTGAC
US28 ORF	GGGCTCGAGATGACACCGACGACGACG	CCCCTCTAGATTACTTGTCGTCGTCGTCCTTGTAGTCGAACCCCTTGTCGTCGTCGTCCTTGTA
UL78 ORF	GCCGCTCGAGATGGGTAAGCCTATCCCTAACCCTCTCCTCGGTCTCGATTCTACGTCCCCTTCTGTGGAGGAGACTAC	CGACTCTAGATCACGTAGAATCGAGACCGAGGAG

*Underlined sequences correspond to restriction sites in the primers: forward, XhoI and reverse, XbaI.
